# Mitochondrial DNA variability of the Polish population

**DOI:** 10.1038/s41431-019-0381-x

**Published:** 2019-03-21

**Authors:** Justyna Jarczak, Łukasz Grochowalski, Błażej Marciniak, Jakub Lach, Marcin Słomka, Marta Sobalska-Kwapis, Wiesław Lorkiewicz, Łukasz Pułaski, Dominik Strapagiel

**Affiliations:** 1 Biobank Lab, Department of Molecular Biophysics, Faculty of Biology and Environmental Protection, University of Łódź, Łódź, Poland; 2 BBMRI.pl Consortium, Wrocław, Poland; 3 Department of Antrophology, Faculty of Biology and Environmental Protection, University of Łódź, Łódź, Poland; 4 Department of Molecular Biophysics, Faculty of Biology and Environmental Protection, University of Łódź, Łódź, Poland; 5 Laboratory of Transcriptional Regulation, Institute of Medical Biology PAS, Łódź, Poland

**Keywords:** Genetics, Molecular biology

## Abstract

The aim of the present study was to define the mtDNA variability of Polish population and to visualize the genetic relations between Poles. For the first time, the study of Polish population was conducted on such a large number of individuals (5852) representing administrative units of both levels of local administration in Poland (voivodeships and counties). Additionally, clustering was used as a method of population subdivision. Performed genetic analysis, included F_ST_, MDS plot, AMOVA and SAMOVA. Haplogroups were classified and their geographical distribution was visualized using surface interpolation maps. Results of the present study showed that Poles are characterized by the main West Eurasian mtDNA haplogroups. Furthermore, the level of differentiation within the Polish population was quite low but the existing genetic differences could be explained well with geographic distances. This may lead to a conclusion that Poles can be considered as genetically homogenous but with slight differences, highlighted at the regional level. Some patterns of variability were observed and could be explained by the history of demographic processes in Poland such as resettlements and migrations of women or relatively weaker urbanisation and higher rural population retention of some regions.

## Introduction

mtDNA analysis has a very important role in the identification of the origin of individuals in a population. It is used especially in population genetics and molecular evolution studies and allows to understand the question of human migration and settlement from different regions of a country or the whole world [1, 2]. Maternally inherited mitochondrial DNA haplogroups indicate the mother line ancestry and have been identified in geographically isolated populations throughout the globe [3]; indicating the human migration and ancestry [4]. Haplogroups from Africa (L0, L1, L2, L3) are found to be the oldest and those which have evolved to European, Asian and Native American ones with geographic migrations and climate adaptations [5]. Nine haplogroups are found to be major in the European population and are as following: H, U, J, T, K, W, I, V and X [6, 7]. The most frequent European haplotypes were classified into HV, U and JT macro-haplogroups forming 90% of population [3]. Several sources indicate the haplogroup H as the most frequent in Europe [8].

mtDNA variability in Polish population was studied in comparison to Russians [1, 2] or as an element of broader group of Slavs [9–11]. Studies with specific attention to administrative division and/or geographic context are still limited. There is no detailed information about mtDNA haplotypes which are characteristic for the representatives of particular voivodeship (województwo) or county (powiat) in Poland. Clustering, as an additional method of grouping of individuals, has also never been used in relation to the Polish population.

National population biobanks and sample repositories store human biological material for the use mostly in genetic research to connect the lifestyle and medical history with genetic traits. Genetic and molecular information associated with the data about the sample donor can also be used in population studies [12]. Furthermore, high-density SNP microarrays, a successful tool to analyse large amounts of genetic data, were used in many population studies to analyse the structure and ancestry of global [13], European [14–16] and individual country populations [17, 18].

The aim of the present study was to determine mtDNA variability of the Polish population, including geographical and historical context. For this purpose, obtained haplotypes of 5852 individuals were classified into major haplogroups and subhaplogroups, and their distribution for units of the first (voivodeship) and second (county) level of local government and administration in Poland was analysed. For the first time, the study of the Polish population was conducted on such a large number of individuals. The gathered data set was then clustered on the basis of genetic information as well as the information about the place of origin, letting us to compare the quite artificial division into voivodeships (*n* = 16) and counties (*n* = 349) with the more natural division into clusters (*n* = 80) which may largely correspond to geographic regions.

## Materials and methods

### Population

**Fig. 1:**
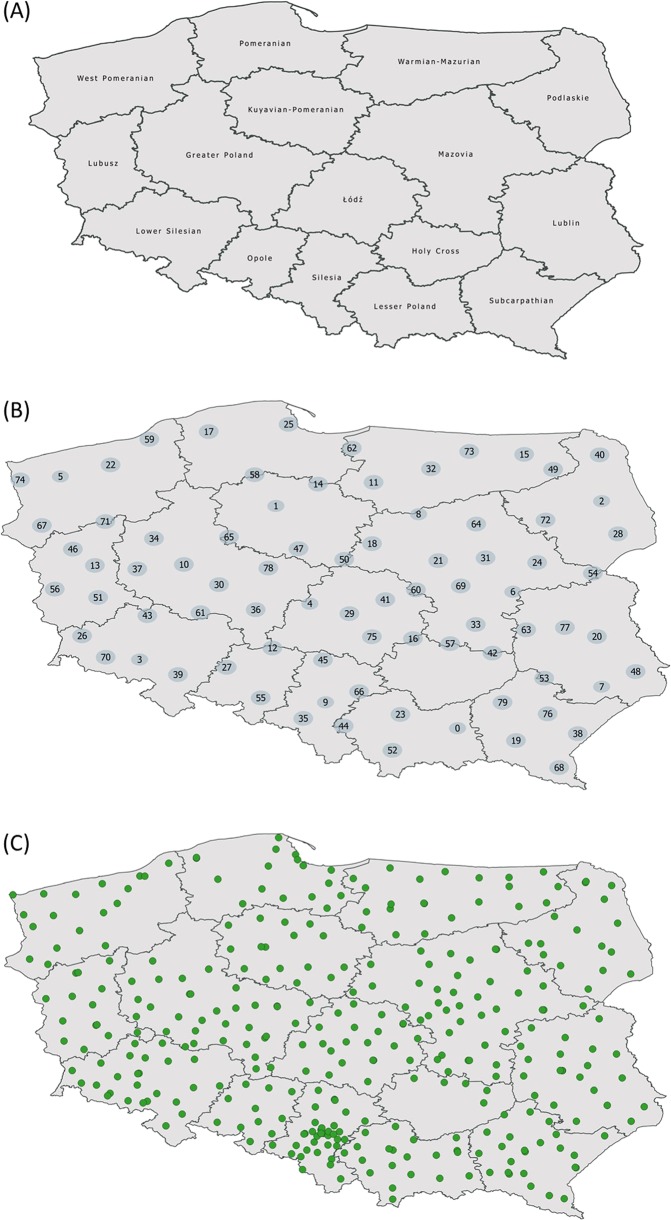
The map showing location of counties with the voivodeships (A), the clusters (B) and population samples (C) used in the following study

The studied population consisted of individuals recruited between 2010 and 2012 within the TESTOPLEK research project. All samples belonged to the POPULOUS collection which is registered since 2013 in the BBMRI catalog [19, 20]. The experimental group included samples taken from 5852 individuals representing administrative units of both levels of local administration in Poland: all 16 voivodeships (Fig. 1 and Fig. S1) and the majority of counties (349 out of total 380—this number includes counties and city counties). Written information about the place of birth and current residence was obtained from each subject. Approval for this study was obtained from the University of Łódź Ethics Review Board. All procedures were performed in accordance with the Declaration of Helsinki (ethical principles for medical research involving human subjects). The full set of results can be obtained at the European Genotype Archive [21] (www.ebi.ac.uk/ega; study accession number, EGAS00001003309).

### Clustering

K-means clustering method applied to spatial coordinates was used to merge individual counties in larger geographic groups (clusters) on the basis of the nearest mean. Each cluster (Fig. 1) is represented by the geographic centre of the cluster and the algorithm converges to stable centroids of clusters [22]. Clustering was performed with Scikit-learn package [23] in Python ver. 3.6.3 [24].

The list of clusters containing the information about the cluster number to which each county was assigned as well as the name of the corresponding geographic region, is gathered in Supplementary Table S1.

### Microarray analysis

Infinium HTS Human Core Exome PLUS microarrays were used to genotype DNA samples for 551,945 SNPs according to the manufacturer’s protocol (Illumina Inc., San Diego, CA, USA). Qualitative analysis was performed to identify outliers and artefacts on the microarray. Samples were excluded if call rate was below 0.94 and if the 10% GenCall parameter was below 0.4. Visual inspection was conducted to investigate the heteroplasmy, which was detected only in a few cases.

### mtDNA typing

Applied microarrays allowed the identification of 323 SNPs (single nucleotide polymorphisms) in mtDNA (Tab. S2)according to recommendations for the description of sequence variants [25]. Quality control procedures were conducted using PLINK software [26]. The homemade script was used to convert raw data obtained in PLINK format for use by Haplogrep software.

### Statistical analysis

Haplogrep software was then used to classify haplotypes into haplogroups and subhaplogroups (Phylotree build 17, http://phylotree.org/tree/index.htm) [27]. Haplogroup frequencies were calculated for every voivodeship and county by counting. The analysis of molecular variance (AMOVA) together with F_ST_ values [28], both for voivodeships and clusters was determined using Arlequin v3.5 software [29]. To visualize the relationships between every voivodeship and every cluster, multidimensional scaling (MDS) analysis was constructed to plot the pairwise genetic distances F_ST_ with cmd scale function in R ver. 3.4.2 [30]. Furthermore, to determine the spatial pattern of genetic divergences (to type the most probable, geographic model of population grouping), SAMOVA (spatial analysis of molecular variance) [31] was done in SAMOVA ver. 2.0 software.

Finally, the geographical distribution of lineages H, U, J, T, HV, K, W, I in Polish population was represented by a surface interpolation map built with QGIS version 2.18.16 [32].

## Results

### Haplogroup and subhaplogroup distribution

**Fig. 2:**
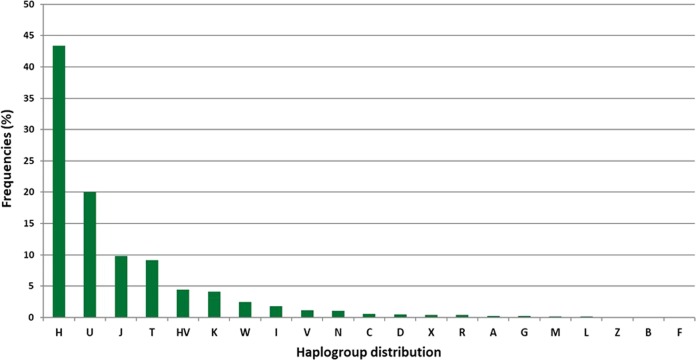
The distribution of mtDNA haplogroups found in Polish population (*N* = 5852 individuals)

21mtDNA haplotypes belonging to haplogroups (Table 1) and 325 belonging to subhaplogroups were found in Polish population (Tab. S3). The most frequent haplotypes were classified into West-Eurasian haplogroups (H, U, J, T), forming 82.38 % of all studied samples (Fig. 2). As expected, haplogroup H was the most frequent in the Polish population (43.42%) (Fig. 2). Among mtDNA subhaplogroups, H1 (15.42%), U5 (12.35%) and J1 (8.34%) showed the highest frequency in the Polish population (Table S3). Asian (C, D, R, A, G, Z, B, F) and African (N, L, M) haplogroups were also found but with very low frequency (Fig. 2). C (0.5%), D (0.46%), X (0.39%), R (0.38%), A (0.26%) haplogroups were quite rare with the frequency lower than 1% (Fig. 2). Haplogroups: L (0.14%), Z (0.09%), B (0.07%), F (0.02%) occurred in Polish population with the lowest frequency (less than 0.15%) and were found only in very few individuals in Greater Poland, Silesian, Pomeranian, Kuyavian-Pomeranian, Lublin, Mazovia, Lesser Poland and Subcarpathian voivodeships (Fig. 2). The haplogroup and subhaplogroup frequencies are presented in Table 1 and Table S3.

**Table 1:** Haplogroup frequencies for Polish population including division into voivodeships (N = 5852 individuals)

Haplogroup frequencies (%)																	
Haplogroup	Total (*n* = 5852)	Lower Silesia (*n* = 317)	Kuyavian – Pomeranian (*n* = 330)	Lublin (*n* = 440)	Lubusz (*n* = 232)	Łódź (*n* = 253)	Lesser Poland (*n* = 311)	Mazovia (*n* = 531)	Opole (*n* = 222)	Subcarpathian (*n* = 410)	Podlaskie (*n* = 232)	Pomeranian (*n* = 412)	Silesia (*n* = 963)	Holy Cross (*n* = 73)	Warmian- Mazurian (*n* = 286)	Greater Poland (*n* = 571)	West Pomeranian (*n* = 269)
A	0.26	0.00	0.30	0.23	0.86	0.00	0.32	0.56	0.45	0.00	0.86	0.00	0.10	0.00	0.35	0.18	0.37
B	0.07	0.00	0.00	0.00	0.00	0.00	0.00	0.00	0.00	0.00	0.00	0.24	0.21	0.00	0.00	0.18	0.00
C	0.55	0.32	0.91	0.00	0.86	0.00	0.64	0.94	0.45	0.98	0.43	0.24	0.52	1.37	0.35	0.53	0.74
D	0.46	0.63	0.61	0.91	0.00	0.00	0.32	0.56	0.45	0.73	0.86	0.49	0.52	0.00	0.70	0.00	0.00
F	0.02	0.00	0.00	0.00	0.00	0.00	0.00	0.00	0.00	0.00	0.00	0.00	0.00	0.00	0.00	0.18	0.00
G	0.21	0.00	0.00	0.00	0.00	1.19	0.00	0.19	0.45	0.49	0.00	0.24	0.10	0.00	0.70	0.18	0.00
H	43.42	45.43	47.27	41.36	43.53	41.50	42.44	43.88	40.99	45.12	44.83	43.69	43.20	42.47	43.36	42.38	42.75
HV	4.46	4.42	6.97	3.86	2.59	5.53	4.18	3.39	4.05	5.12	3.88	3.64	5.71	2.74	2.10	5.43	2.97
I	1.76	3.47	2.12	1.82	1.72	0.79	1.61	2.07	2.70	0.73	0.86	2.43	1.14	4.11	2.45	1.58	1.49
J	9.77	10.41	6.67	9.32	9.48	13.44	9.65	9.79	9.01	8.78	10.78	7.28	10.70	17.81	11.19	11.03	5.95
K	4.07	4.10	3.33	4.09	3.02	2.37	2.89	4.52	2.25	5.12	4.31	6.07	3.32	2.74	3.85	4.73	6.32
L	0.14	0.00	0.00	0.00	0.00	0.00	0.64	0.00	0.00	0.00	0.00	0.00	0.31	0.00	0.00	0.53	0.00
M	0.15	0.00	0.00	0.00	0.00	0.00	0.64	0.00	0.00	0.00	0.00	0.24	0.21	0.00	0.00	0.70	0.00
N	1.06	1.26	0.30	0.68	0.43	0.79	1.61	1.13	0.45	1.22	0.00	1.21	1.56	2.74	2.10	0.53	1.12
R	0.38	0.00	0.00	0.45	0.43	1.98	0.32	0.19	0.00	0.49	1.29	1.21	0.10	0.00	0.00	0.00	0.37
T	9.13	10.09	8.18	8.41	12.50	6.72	7.72	9.23	9.91	9.76	11.64	9.22	8.41	8.22	11.54	7.88	10.04
U	20.06	16.40	19.70	24.55	20.26	21.74	21.86	19.96	24.77	18.29	17.24	17.48	19.83	16.44	17.13	19.79	24.54
V	1.16	0.95	0.91	0.91	1.29	1.98	0.64	0.94	1.35	0.00	0.43	1.46	1.66	1.37	1.75	1.75	0.37
W	2.41	1.58	2.12	2.73	2.59	1.98	3.54	2.26	2.25	2.44	2.59	4.61	1.97	0.00	2.45	1.93	2.23
X	0.39	0.95	0.30	0.45	0.43	0.00	0.96	0.19	0.45	0.24	0.00	0.24	0.42	0.00	0.00	0.53	0.74
Z	0.09	0.00	0.30	0.23	0.00	0.00	0.00	0.19	0.00	0.49	0.00	0.00	0.00	0.00	0.00	0.00	0.00

### Voivodeship comparison

**Fig. 3:**
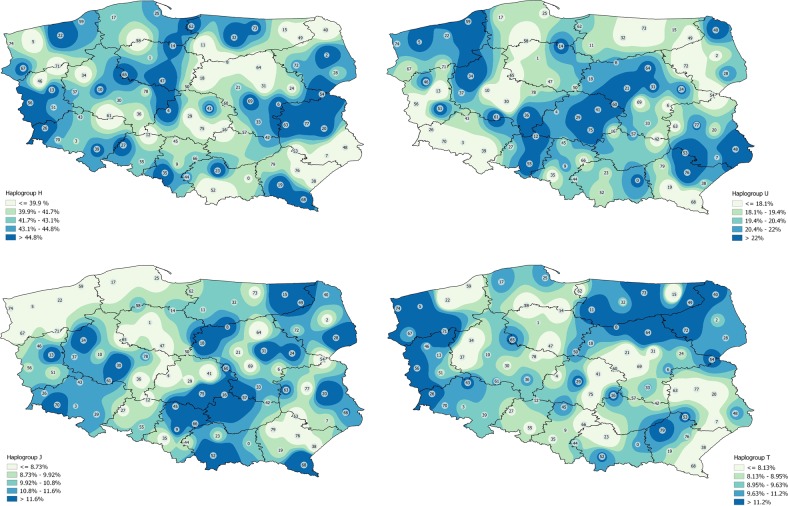
Interpolation maps for the main haplogroups (H, U, J, T) observed in the Polish population

**Fig. 4:**
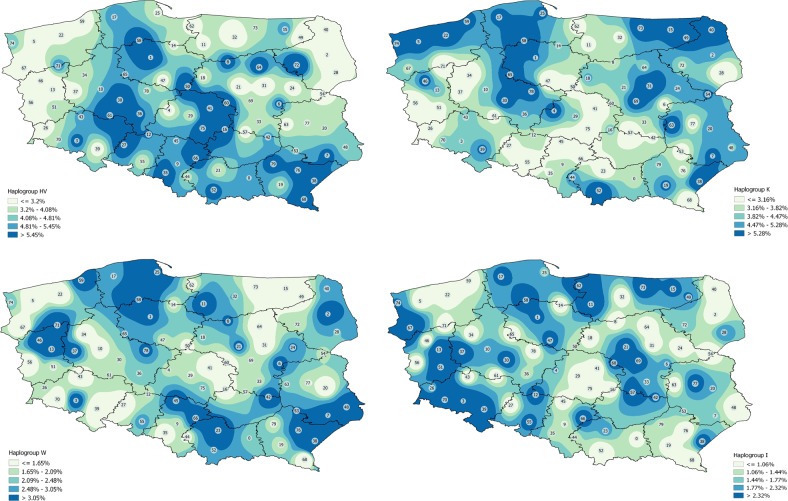
Interpolation maps for the other main haplogroups (HV, K, W, I) observed in the Polish population

Four voivodeships, the Greater Poland, Silesian, Łódź, and Lower Silesian ones, reveal similar structure to Poland’s average in terms of relative frequency of the six major haplogroups (H, U. J, T, HV, K). The highest number of haplogroups (*n* = 19) was observed in Silesian voivodeship while the lowest (*n* = 10) was observed in Holy Cross voivodeship. However, they were represented by as the largest and the smallest sample number; 963 and 72, respectively. Among the voivodeships with the sample number between 200 and 500, 17 haplogroups were observed in Lesser Poland while only 12 in Łódź voivodeship. Analysis based on Pearson’s Chi-square test was performed to assess the differences between voivodeships infrequencies of 10 main haplogroups (Table S4–S13). The obtained results of Pearson’s Chi-square test pushed us to look more closely at the differences between regions. Therefore, interpolation analysis was performed for the frequencies of eight main haplogroups to show their distribution across Poland. Illustration of the frequencies of haplogroups on the map of Poland using interpolation method allowed us to underline the differences between regions. Different pattern of distribution of eight main haplogroups was observed for every voivodeship. However, observed differences were on a relatively low level (Figs 3 and  4).

### Genetic variability

To define differentiation among Polish population in terms of the similarities and differences between voivodeships, paired F_ST_ analysis were performed. All F_ST_ estimates were positive but low and ranged from 0.00011 to 0.02045 (Table S14). The highest, statistically significant differences were observed between F_ST_ values calculated for Holy Cross and West Pomeranian (F_ST_ = 0.02045; *p* = 0.0001) as well as for Holy Cross and Pomeranian (F_ST_ = 0.01509; *p* = 0.008). Furthermore, Holy Cross was the only voivodeship with F_ST_ values higher than 0.01; the differences concerned also Lesser Poland (F_ST_ = 0.01483; *p* = 0.0065); Subcarpathian (F_ST_ = 0.01416; *p* = 0.0114); Lublin (F_ST_ = 0.01323; *p* = 0.0167); Opole (F_ST_ = 0.01266; *p* = 0.0345) and Kuyavian-Pomeranian (F_ST_ = 0.0122; *p* = 0.0234) (Fig. S2 and Table S14).

Łódź was another unit where differences (significant F_ST_ values) were observed for the same voivodeships as in Holy Cross comparison. However, F_ST_ values were lower in each case: Lesser Poland (F_ST_ = 0.00431; *p* = 0.0445); Subcarpathian (F_ST_ = 0.00528; *p* = 0.0104); Lublin (F_ST_ = 0.00393; *p* = 0.0475); Opole (F_ST_ = 0.00528; *p* = 0.0463) and Kuyavian-Pomeranian (F_ST_ = 0.000477; *p* = 0.0335). As in the case of Holy Cross, the highest F_ST_ values for this region were observed with West Pomeranian (F_ST_ = 0.00874; *p* = 0.0015) and Pomeranian (F_ST_ = 0.00673; *p* = 0.0026) (Fig. S2 and Tab. S14).

West Pomeranian was the third unit with statistically significant differences observed in calculated F_ST_ values for numerous voivodeships: apart from the above-described Holy Cross and Łódź, also Warmian-Mazurian (F_ST_ = 0.00753; *p* = 0.0046); Podlaskie (F_ST_  =  0.00607; *p* = 0.0278); Lower Silesian (F_ST_ = 0.00541; *p* = 0.0189); Lesser Poland (F_ST_ = 0.00413; *p* = 0.05) and Silesian (F_ST_ = 0.00393; *p* = 0.0182) (Fig. S2 and Table S14).

Additionally, paired F_ST_ analysis was also performed for clusters. In this case, F_ST_ estimates were positive, quite low and ranged between 0 and 0.07907 (Table S15). The highest F_ST_ estimates were identified between clusters: **71** (Choszczno and Drezdenko counties) and **41** (Łowicz, Brzeziny and Tomaszów counties) (F_ST_ = 0.07907, *p* = 0.00564) and also between clusters: **64** (Mazovia, Ostrołęka counties) and **23** (Kraków county) (F_ST_ = 0.07038; *p* = 0.0003) (Fig. S3 and Table S15). Furthermore, the largest number of statistically significant F_ST_ estimates were observed for the following clusters **23**, **34** (Czarnków and Szamotuły counties), **41**, **59** (Koszalin and Sławno counties) and **64** among many others (Fig. S3 and Tab. S15).

Detailed information about F_ST_ values calculated for all voivodeships and clusters are gathered in Tables S14 and S15 (in Supplementary materials) and are presented on Figure S2 and S3, respectively.

A MDS plot, on the basis of the pairwise F_ST_ values, was constructed to visualize the relationships between voivodeships (Fig. S4). A group including Lublin, Lesser Poland, Greater Poland, Mazovia, Silesia, Kuyavian-Pomeranian, Opole, Lubusz, Subcarpathian, Lower Silesian and Podlaskie was observed while Łódź, West Pomeranian, Pomerania, Warmian-Mazurain, Holy Cross were observed as separate. Another MDS plot was constructed to visualize the relationships between generated clusters (Fig. S5). In this case, a large group of almost all clusters was observed together, while clusters number: **16** (Rawa and Opoczno regions), **18** (Płock and Sierpc Lands), **23** (Kraków county**), 31** (Mazovia region, comunies Wołomin and Wyszków), **34** (northern Greater Poland, Szamotuły and Trzcianków county), **41** (Łowicz, Brzeziny and Tomaszów counties in Łódź voivodeship), **47** (Western Kuyavia), **49** (Ełk and Grajewo regions), **64** (northern Mazovia) and **71** (Choszczno and Drezdenko counties) were outside of this group.

### AMOVA

Analysis of molecular variance based on the mtDNA sequences reveals that most of the variation occurs within populations when voivodeships were taken into account (99.78%; *p* = 0.01075). Only a small proportion of total variance was attributed to variation among groups also in the case of voivodeships (0.21%; *p* = 0.01075) (Table 2). Analysis of molecular variance computed for cluster populations also reveals that most of the variation occurs within populations (99.09%; *p* = 0.00109). Only a small proportion of total variance was attributed to variation among clusters (0.91%; *p* = 0.00109) (Table 2).

**Table 2:** Analysis of molecular variance (AMOVA) accounting for all voivodeships and clusters

Grouping method	Percentage of variation		Fixation index (F_ST_)
	Among populations	Within populations	
Voivodeships	0.21	99.79	0.00214*
Clusters	0.91	99.09	0.00908**

*p = 0.01075

**p  =  0.00109

### SAMOVA

Analysis of the molecular variance conducted in SAMOVA ver. 2.0 software, based on the mtDNA SNPs and aimed at determining the most probable number of genetically different population groups, showed that the maximal number of significantly divergent groups is 33. The highest variance among groups was observed when the population was divided into 2 groups (cluster no. 64 separated from the rest of clusters, variance among groups = 2.41%; *p* < 0.00001). With the ncreasing number of groups we could observe downward sloping trend with fluctuations, so we could identify local maxima (2, 4, 7, 11, 15, 18, 21, 23, 25, 29 and 31 groups). When dividing into groups corresponding to local maxima, the following clusters were separate: no. 64 (7 times), no. 34 (6 times) and no. 71 (5 times) while cluster 37 was grouped with 59 (5 times).When dividing into the maximum number of 33 groups, the variance among groups was equal to 0.32% (*p* < 0.00001).

## Discussion

mtDNA variability in Polish population was previously studied in comparison to Russians [1, 2] or as the element of broader group of Slavs [9–11]. In the current study, an attempt to completely describe mtDNA variability and genetic connections for Polish population was made, based on a large group of individuals (5852) and including administrative unit clustering as an additional method of population dividing for increased geographical relevance. Analysing the frequencies of haplogroups, H was found to be the one most often occurring in the Polish population. It is consistent with the findings of Grzybowski et al. [9] and Mielnik-Sikorska et al. [11]. An interesting analysis of haplogroup and subhaplogroup distribution was done by Malyarchuk et al. [1] but we can compare our results only to the main findings for the entire Polish population without the division into regions. The cited study of Polish population showed 45.2 % frequency [1], which is almost identical to our findings. Similarly, in the case of U, J, T, K and W haplogroups, frequencies obtained in the current study were practically the same compared to Malyarchuk et al. [1], whose study was based on the analysis of 436 individuals from Kuyavian-Pomeranian region. The only difference was observed in the case of HV haplogroup. Malyarchuk et al. [1] identified 1% frequency of this haplogroup while in our study it was 4.46 %. The number of individuals can be an explanation, as in the case of rare haplogroups, the size of studied samples has a great importance. Our findings are also consistent with other studies of European population [8, 33] as well as individual countries such as: Spain [34, 35], Portugal [36] with Azores [37], islands of North Atlantic [38], Sardinia [39] and Russia [1, 2], where haplogroup H was also indicated as the most frequent.

Most of the voivodeships in Poland reveal divergent patterns of major haplogroup frequencies, which differ from the values for Poland in general. In literature data, description of regional populations of Poland basing on the mtDNA haplogroup distribution can be found only for selected regions, such as: Gdańsk, Kashubia, Suwałki, Upper Silesia [9] and Podhale [11]. In the case of haplogroup H, our results (compared at the level of appropriate administrative units, i.e., voivodeships or counties) were consistent with literature for all studied regions except Podhale where the frequency was around 30% [11] while in our study (Tatra county) it was 19,5%. In Gdańsk region, frequencies of the 6 most common haplogroups obtained in the studies of Grzybowski et al. [9] were almost the same as in the current study:

Relating to the studies about Ashkenazi maternal lineages [40] and mitochondrial markers of Jewish ancestry [41] and analysing proposed motifs to define four major Ashkenazi founder clusters (K1a1b1a, K1a9, K2a2a and NH1b1), we could not present their occurrence within the Polish population because of the lack of polymorphic sites (16093-16176-16223-16224-16234-16311-16519) on the microarray used. Only one site from the proposed motif was present (16145). Grzybowski et al. [9] found K1a1b1a lineage in individuals from Gdańsk region and Upper Silesia, based on the specific mtDNA motif.

Interestingly, the frequency of L haplogroup, one of the rarest in Europe, observed in the current study and the study of Mielnik-Sikorska et al. [11] was similar for Podhale region (1% vs. 3%). L1b is the most common African clade in Europe; [42] in the studies of Mielnik-Sikorska et al. [11], L1b1a8a and L2a subclusters were identified among Polish individuals, with the presence of L2a1 haplotype ascribed to Ashkenazi Jewish influences. In this study, both haplotypes (L1b1 and L2a1) were found in individuals from different regions of Poland: 4 individuals with L1b1 from Upper Silesia and 3 individuals with L2a1 haplotype from Gorlice and Częstochowa counties. We additionally identified L2e and L3e subclades: 1 individual with L2e from Nowy Tomyśl county and 2 individuals with L3e from Poznań. Interestingly, L0 is the most common haplotype in East Africa, the Near East and Arabian Peninsula [43]. In the current study, L0a1a was found in 2 individuals from Tatra county. In the current study, we focused on the genetic relationships and regional connections, omitting a detailed subhaplogroup analysis. However, the frequencies were calculated and H1 (15.42%), U5 (12.35%) and J1 (8.34%) were observed as the most frequent subhaplogroups in Polish population; this is also in agreement with the studies of Grzybowski et al. [9] (Table S2).

As mentioned above, the Polish population was the subject of the genetic research, but only in comparison to broader groups of Slavs or Europeans. Grzybowski et al. [9] made a genetic analysis, based on haplogroup frequencies, of four populations from Poland (Suwałki, Gdańsk regions, Kashubia and Upper Silesia) in comparison to selected populations of Russia. Suwałki was indicated as the most divergent region, separated from remaining Polish populations and grouped together with northwestern Russians. In our study, Suwałki region was treated as a separate cluster (cluster no. 40) consisting of Augustów, Sejny and Suwałki counties, or as a part of Podlaskie voivodeship. At the cluster level, statistically significant differences (F_ST_ distances) were observed for Suwałki region and Kraków Land (F_ST_ = 0.026) and Kuyavia (0.029). However, MDS analysis did not show this region as significantly divergent. When Suwałki region was treated as a part of Podlaskie voivodeship, the only difference, based on F_ST_ values, was observed with Western Pomerania. Nevertheless, MDS analysis did not show Podlaskie as a divergent voivodeship. In our study, raw data based on SNP was used to compute F_ST_ values, while Grzybowski et al. [9] used haplogroup frequencies. It could be another reason for difficulties in the comparison of results. Furthermore, Malyarchuk et al. [2] found slight differences between Polish, Russian and Estonian populations, investigated by AMOVA. On the other hand, their MDS plot showed Poles as separate from the rest of the studied populations. It is worth adding that in this case, Polish population was represented only by individuals from Kuyavian-Pomeranian region. In our study, Kuyavian-Pomerania was analysed as a separate voivodeship but also as two separate clusters (cluster no. 47 and 50) corresponding to the Western and Eastern Kuyavia region, respectively. When Kuyavia-Pomeranian was treated as a whole voivodeship, statistically significant differences were observed only for F_ST_ values between this region and Holy Cross and Łódź voivodeships. However, F_ST_ values were still very low and cannot prove genetic separation, which is confirmed by the MDS plot, where Kuyavian-Pomeranian was grouped together with the rest of voivodeships. At the level of clusters, the situation is different. Cluster no. 47 corresponding to Western Kuyavia (including Radziejów, Aleksandrów, Koło and Inowrocław counties) was observed as different to many other regions of Poland. F_ST_ values ranged between 0.0140 and 0.0660; all were statistically significant. The highest F_ST_ value was noted between Western Kuyavia and Northern Mazovia (cluster no. 64 consisting of Maków, Ostrołęka, Przasnysz and Pułtusk counties). MDS plot clearly illustrated the separation of this region from the rest of Poland. Interestingly, Eastern Kuyavia (cluster no. 50 consisting of Włocławek, Lipno, Kutno and Gostynin counties) did not show many differences to other clusters, based on F_ST_ values. The only two statistically significant differences were observed for cluster no. 34 (Czarnków, Szamotuły counties) and 59 (Koszalin and Sławno counties) compared to Eastern Kuyavia, but F_ST_ values were rather low. MDS plot confirms that Eastern Kuyavia is not genetically separate within the Polish population.

The history of Poland, especially in the last century, was marked by extensive human resettlements that took place during and shortly after the Second World War (WWII). The reasons of massive migration of Poles are following: the exile and internal exile of Poles during the September campaign at the beginning WWII; displacement of Poles from areas annexed to USSR (the Union of Soviet Socialist Republics); deportation for forced labour under German rule during World War II; Polish population transfers after WWII connected with the change of borders; economical migrations [44]. All of these have caused the destruction of social relations, but on the other hand, allowed to form a well-mixed and homogeneous population. The homogeneity of the Polish population was mentioned before in the studies of Płoski et al. [45], Kayser et al. [46], Woźniak et al. [47] and Rębała et al. [48], however, results were based only on the analysis of Y chromosome. The results of this study complement the description of the Polish population and confirm that our population is homogenous as far as mtDNA variability is concerned. Our study showed that most of the molecular variation based on the mtDNA sequences occurs within the population at large and a very low variation was detected among subpopulations, both when voivodeships and clusters were taken for analysis. Despite this homogeneity, some patterns of variability (separate voivodeships and clusters) are observed and can be explained by the history of demographic processes in Poland. West Pomeranian and Warmian-Mazurian voivodeships were observed as outliers in the Polish population, which could confirm their genetic separateness caused by resettlements and migrations of women. These voivodeships were settled after the Second World War by people inhabiting Kresy (Eastern Borderlands of Poland). West Pomeranian was settled mostly by people from Baranowicze, Pińsk and Kowel regions (now Belarus and Ukraine) while Warmian-Mazurian was settled by Poles living in Vilnius region (now Lithuania) [44]. However, in the current study, Holy Cross and Łódź voivodeships were found to be the most separate, which is not reflected in the history of the migration of Poles. These voivodeships are quite native in their population composition and have not been the areas of massive migrations. In this case, the reason for separation must be different, but is probably also connected with demographic processes occurring in this part of Poland, such as relatively weaker urbanisation and higher rural population retention. Furthermore, detailed analysis of clusters showed that only a few of them located within those voivodeships were statistically separate. Thus, it cannot be proven that migration was the reason for genetic separation.

For the first time, clustering, a method of population subdivision, was used to define the genetic relationships within the Polish population. Additionally, the administrative division of Poland was overlaid on genetic separation in order to present the most complete view of Polish society. Furthermore, for the first time, the study of the Polish population was conducted on such a large number of individuals (5852). All of this makes it difficult to find similar studies to directly relate our findings to results of others. There were important differences in analysis based purely on administrative division and on geographical clustering, which is expected, showing that a large dataset makes it possible to perform a deeper and more relevant analysis.

Our comprehensive analysis of mtDNA variability, based on the data from 5852 individuals, allowed us to describe the mtDNA variability of Polish population and genetic relations between Poles. It gives a better insight into mtDNA variability in Poland, with detailed administrative divisions and geographical regionalization. A complete genetic analysis including all voivodeships and most counties of Poland has been performed for the first time. Poles are characterized by the main West Eurasian mtDNA haplogroups, but relatively minor genetic differences observed on the level of voivodeships and clusters may indicate historical and cultural influences. Although the level of differentiation within the Polish population was found to be low, the existing genetic differences can be explained well with geographic distances. Using a large set of data, it was shown that Poles can be considered as genetically homogenous but with slight differences, highlighted at the regional level. The structure of our study allowed us to confirm that intrastate administrative divisions are artificial formations and do not reflect the genetic diversity of specific populations. Spatial information-based clusters are more adequate and in similar studies, researchers should consider grouping available samples based on geographic location, enhancing the quality of analysis in comparison to division into voivodeships and counties. The following study was based only on mitochondrial markers, which can illustrate gene flow in the maternal line. Therefore, conclusions can be drawn exclusively about migrations and settlements of women. Certainly, the present survey could be the basis for further research relating to the historical context of human migration or resettlements, when expanded with an analysis of chromosome Y.

## Supplementary information

Tab. S1

Tab. S2_haplotypes

Tab. S2_markers

Tab. S3

Tab. S4

Tab. S5

Tab. S6

Tab. S7

Tab. S8

Tab. S9

Tab. S10

Tab. S11

Tab. S12

Tab. S13

Tab. S14

Tab. S15

Fig. S1

Fig. S2

Fig. S3

Fig. S4

Fig. S5
