# Supplementary figures and images for: Mitochondrial DNA variability of the Polish population
Source: Eur J Hum Genet. 2019 Mar 21;27(8):1304–14. doi: 10.1038/s41431-019-0381-x (PMC6777467; doi:10.1038/s41431-019-0381-x)

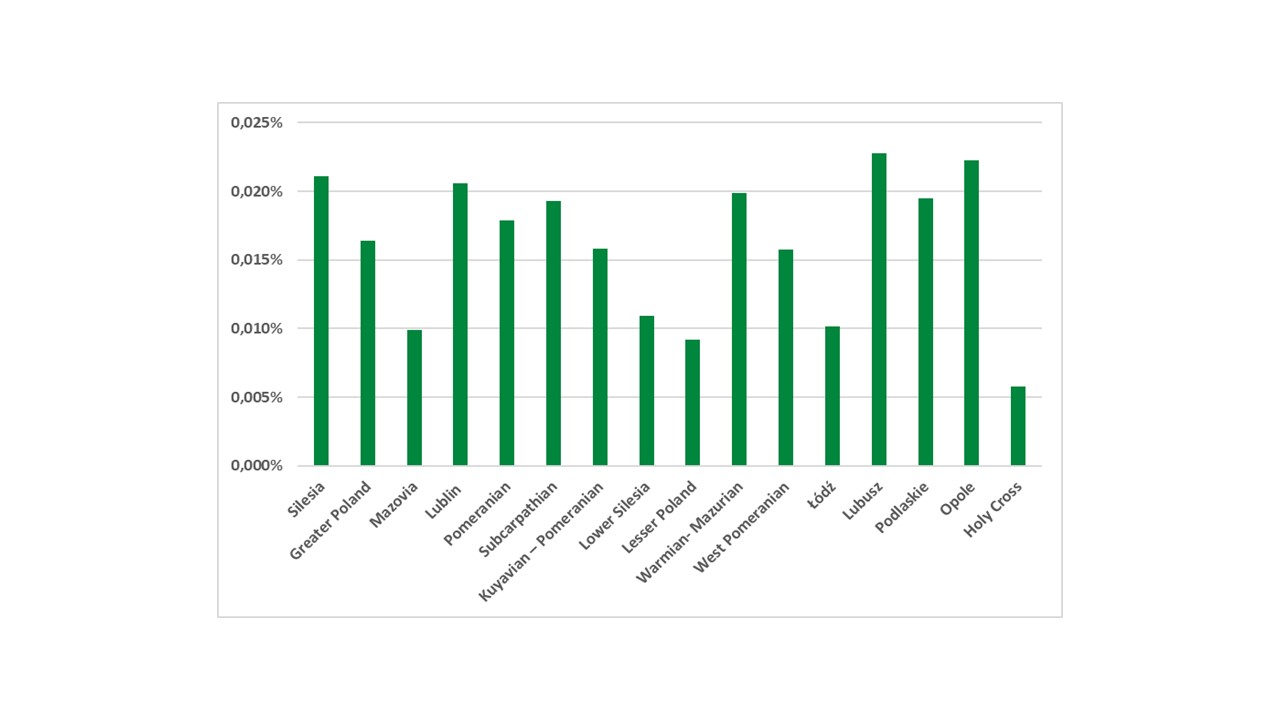

Supplement: Supplementary file 17 — Fig. S1 [file 41431_2019_381_MOESM17_ESM.jpg]

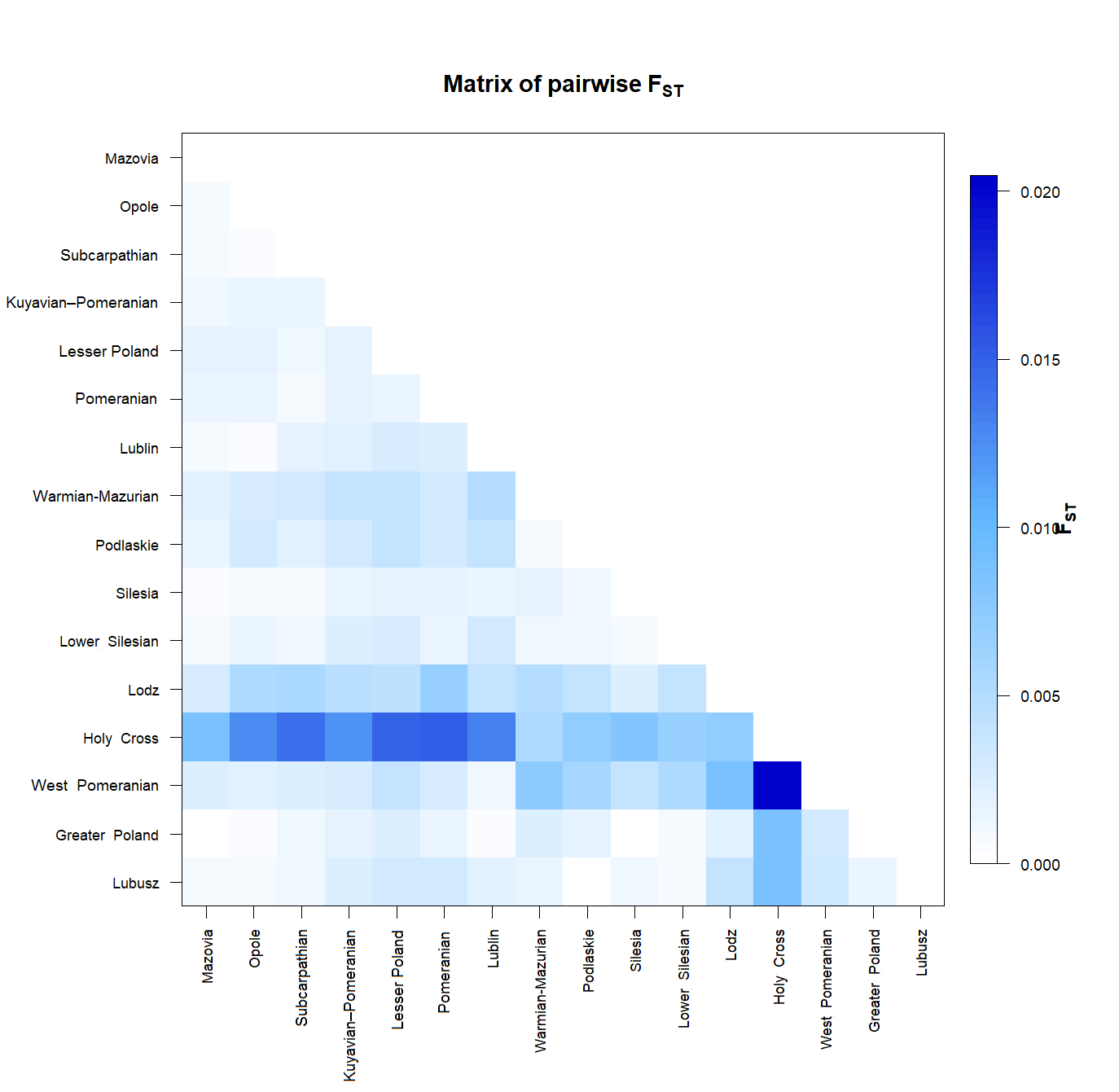

Supplement: Supplementary file 18 — Fig. S2 [file 41431_2019_381_MOESM18_ESM.png]

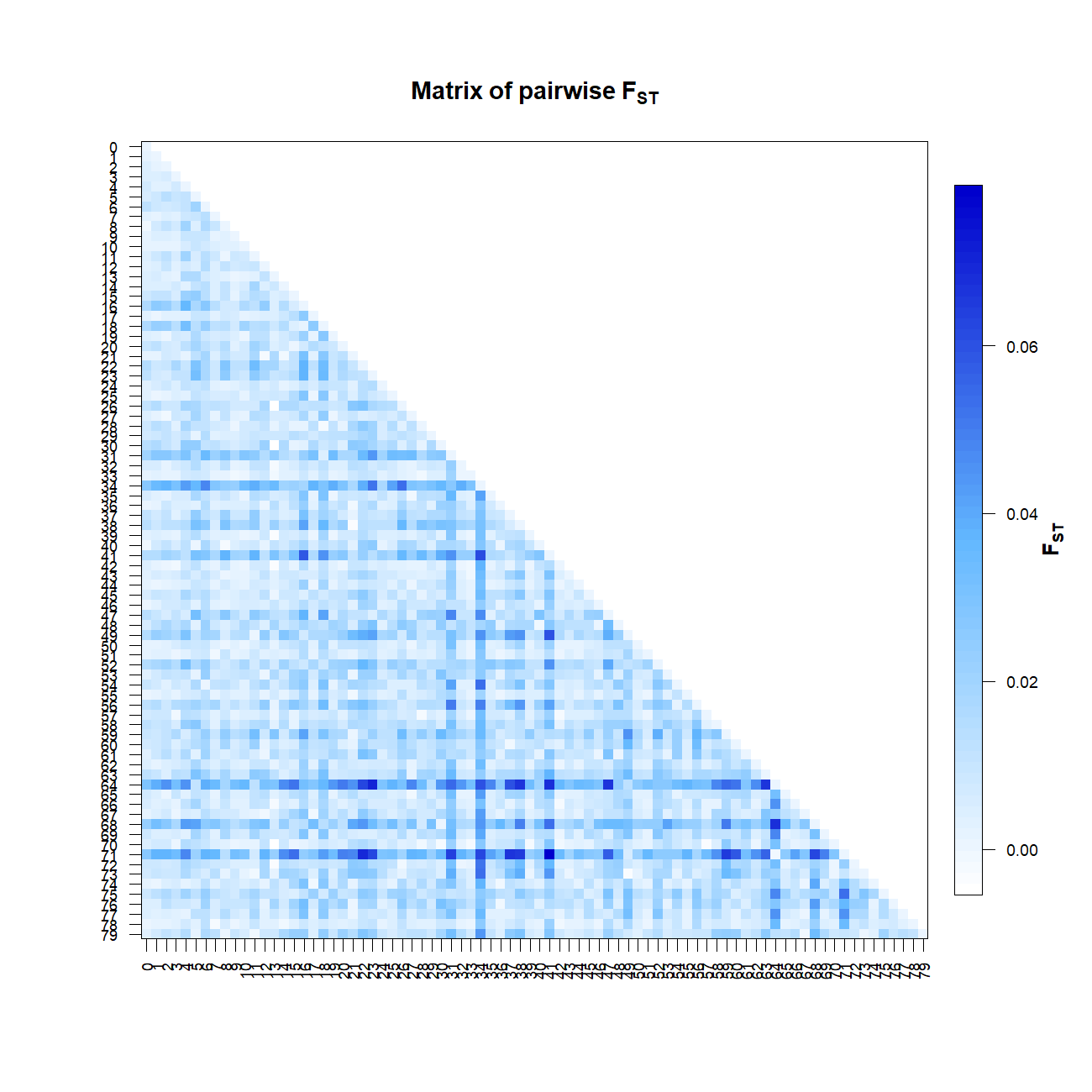

Supplement: Supplementary file 19 — Fig. S3 [file 41431_2019_381_MOESM19_ESM.png]

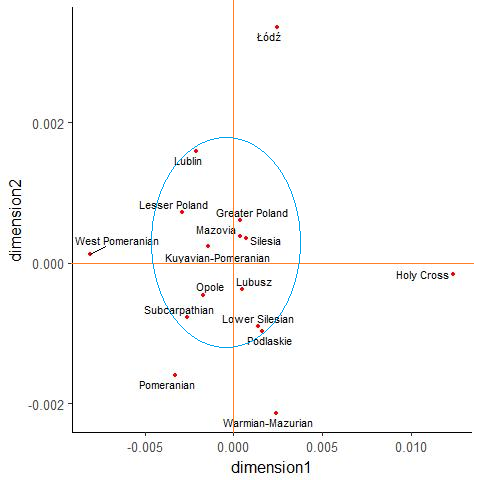

Supplement: Supplementary file 20 — Fig. S4 [file 41431_2019_381_MOESM20_ESM.png]

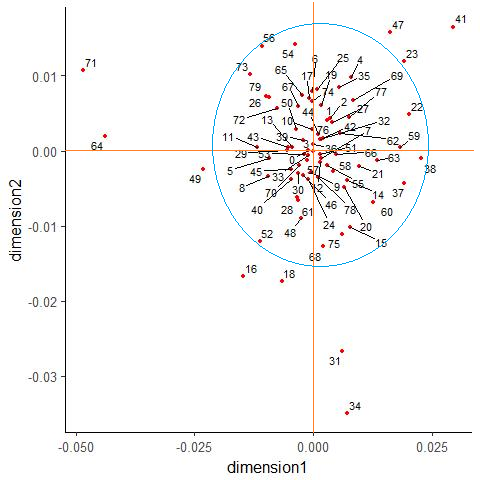

Supplement: Supplementary file 21 — Fig. S5 [file 41431_2019_381_MOESM21_ESM.png]
